# Spinacetin Suppresses the Mast Cell Activation and Passive Cutaneous Anaphylaxis in Mouse Model

**DOI:** 10.3389/fphar.2018.00824

**Published:** 2018-07-30

**Authors:** Ning Ji, Shunli Pan, Chen Shao, Yufen Chen, Zhe Zhang, Ran Wang, Yuling Qiu, Meihua Jin, Dexin Kong

**Affiliations:** ^1^Tianjin Key Laboratory on Technologies Enabling Development of Clinical Therapeutics and Diagnostics, School of Pharmacy, Tianjin Medical University, Tianjin, China; ^2^Pharmacy Department, Tanggu Hospital of Infectious Diseases of Tianjin Binhai New Area, Tianjin, China

**Keywords:** spinacetin, mast cell, IgE/Ag, Syk, PCA

## Abstract

We previously reported the anti-inflammatory and anti-asthmatic activities of the extract of the *Inula japonica* Thunb. Aiming for discovery of a novel anti-inflammatory compound, we isolated spinacetin from the extract and investigated its *in vitro* and *in vivo* anti-inflammatory effect and the related mechanism. Effect of spinacetin on the Syk signaling pathway was studied in bone marrow-derived mast cells (BMMCs), and that on the nuclear factor-κB (NF-κB) and mitogen-activated protein kinases (MAPKs) was investigated in Rat basophilic leukemia (RBL)-2H3 cells and human mast cell line (HMC-1). The *in vivo* anti-inflammatory activity was assessed with passive cutaneous anaphylaxis (PCA) reaction assay. Spinacetin significantly inhibited the release of histamine, and production of inflammatory mediators such as leukotriene C_4_ (LTC_4_) and interlukin-6 (IL-6) in IgE/Ag stimulated BMMCs. Analysis of the signaling pathways demonstrated that spinacetin inhibited activation of Syk, linker of activated T cells (LAT), phospholipase Cγ (PLCγ), cytosolic phospholipase A_2_ (cPLA_2_), MAPKs, Akt/NF-κB, and intracellular Ca^2+^ mobilization but with no effect on Fyn and Lyn. On the other hand, spinacetin suppressed IgE/Ag-induced activation of RBL-2H3 cells with inhibition against phosphorylation of extracellular signal regulated-protein kinase (ERK), c-Jun-NH_2_-terminal kinase (JNK), p38 MAPKs, PLCγ, translocation of cPLA_2_, and Akt/IκBα/NF-κB signal. However, spinacetin had no effect on PMA and A23187-induced activation of HMC-1. Furthermore, oral administration of spinacetin dose-dependently attenuated IgE/Ag-mediated PCA reaction in mouse model. Taken together, spinacetin showed the activities in preventing inflammatory processes, which might be at least partially attributed to the abolishment of Syk-dependent activation of IgE/Ag-mediated mast cells.

## Introduction

Mast cell is known to contribute to allergic diseases such as asthma, rhinitis, urticaria, allergic conjunctivitis, and anaphylaxis (Modena et al., 2016). Mast cells are important effector cells of IgE-mediated immunological and allergic inflammatory reactions (Siraganian, 2003). The aggregation of high affinity IgE receptors (Fc𝜀RI) on mast cells via antigen (Ag) triggers the release of various preformed mediators (including histamine and proteases), newly synthesized lipids including LTs, prostaglandins (PGs), and many inflammatory cytokines such as IL-6, tumor necrosis factor (TNF)-α (Kopec et al., 2006). These mediators play pivotal roles in allergic and inflammatory reactions.

Ag induced crosslinking of Fc𝜀RI activates the Src family tyrosine kinase Lyn, which phosphorylates the immunoreceptor tyrosine-based activation motifs (ITAMs) of Fc𝜀RI and Syk following ITAM binding (Kalesnikoff and Galli, 2008). Aggregation of Fc𝜀RI also rapidly activates Fyn, which phosphorylates the Grb2 associated binding protein 2 (Gab2) to activate the phosphoinositide 3 kinase (PI3K) pathway (Kalesnikoff and Galli, 2008). Activated Syk leads to phosphorylation of LAT. Upon phosphorylation, these proteins serve as scaffolds for multimolecular signaling complexes comprising various cytosolic adapter molecules such as Gads, Grb2, and the signaling enzyme PLCγ (Metcalfe et al., 2009). Activated PLCγ catalyzes the hydrolysis of the PIP_2_ to inositol trisphosphate (IP3) and diacylglycerol (DAG)-second messengers that liberate intracellular Ca^2+^ from internal stores and activate protein kinase C (PKC), respectively (Siraganian, 2003). The above interactions lead to mast cell degranulation, eicosanoid generation, and cytokine production (Vig and Kinet, 2009). Grb2-bound Son of Sevenless (SOS) homolog activates Ras, which consequently initiates the mitogen activated protein kinase (MAPKs) pathway, MAPKs, including ERK, JNK, and p38 (Tkaczyk and Gilfillan, 2001; Anderson, 2006), regulates cPLA_2_ and therefore promotes generation of arachidonic acid, PGs and LTs (Tkaczyk and Gilfillan, 2001). PI3K promotes phosphorylation of Akt, leading to activation of NF-κB and production of cytokines (Kim et al., 2008).

**FIGURE 1:**
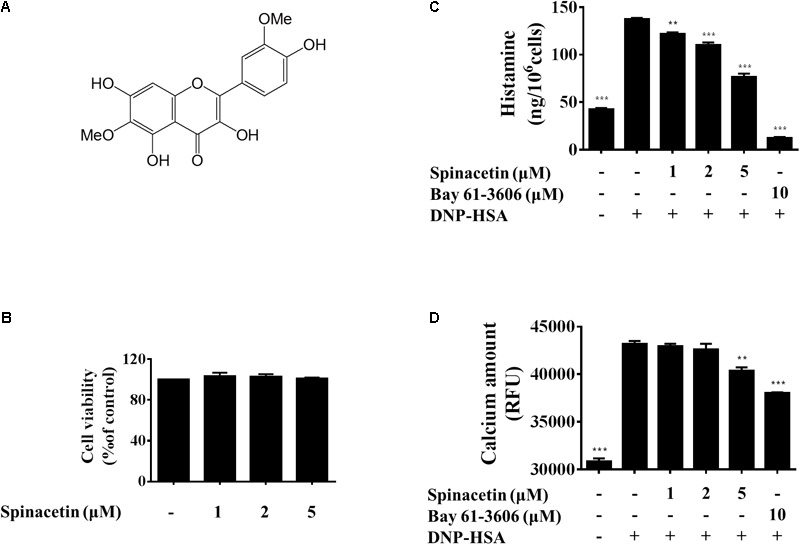
Effect of spinacetin on the degranulation and Ca^2+^ mobilization in IgE/Ag-stimulated BMMCs. **(A)** Chemical structure of spinacetin. **(B)** Effect of spinacetin on cell viability. BMMCs were treated in the absence or presence of spinacetin (1, 2, and 5 mM) for 8 h. Cell viability was determined by MTT assay. **(C)** IgE-sensitized BMMCs were pre-treated with spinacetin or Bay 61-3606 for 1 h, and then stimulated with DNP-HSA for 15 min. The amount of histamine released into the culture media was measured using ELISA. **(D)** IgE-sensitized BMMCs were pre-incubated with FluoForte TM dye-loading solution for 1 h, then treated with spinacetin or Bay 61-3606 for 1 h. The fluorescence was measured after stimulation with DNP-HSA for 5 min. Bay 61-3606 was used as positive control. The data show the mean ± SEM of three independent experiments. ^∗∗^*P* < 0.01 and ^∗∗∗^*P* < 0.01, compared with the cells with IgE/Ag stimulation but without spinacetin treatment.

Spinacetin (**Figure 1A**) is a flavonoid isolated from *Inula japonica* Thunb (Zhu et al., 2014). In our previous study, the extract of *Inula japonica* showed anti-inflammatory and anti-asthmatic activities (Park et al., 2011; Lu et al., 2012). It was reported that spinacetin reduces prostaglandin E_2_ level in macrophages (Moscatelli et al., 2006), and our group found that spinacetin inhibits LTC_4_ synthesis and degranulation in c-Kit ligand induced mast cells (Zhu et al., 2014). However, the anti-inflammatory effect of spinacetin on IgE/Ag-mediated mast cells and anaphylaxis has not been reported yet. Anaphylaxis is a severe systemic reaction closely related to mast cell activation (Akin, 2015). Therefore, we recently evaluated anti-allergic effect of spinacetin and its related molecular mechanism in mast cells and PCA models.

## Materials and Methods

### Reagents

RPMI1640, fetal bovine serum (FBS) and the enhanced chemiluminescence (ECL) Western blot detection reagent were purchased from Thermo Fisher Scientific Inc. (Waltham, MA, United States). Mouse anti-dinitrophenyl (DNP) IgE was purchased from Sigma Chemicals (St. Louis, MO, United States). DNP-human serum albumin (HSA) was from Biosearch Technologies (Petaluma, CA, United States). The antibodies specific for phospho-ERK1/2, ERK1/2, phospho-p38, p38, phospho-JNK1/2, JNK1/2, phospho-PLCγ, phospho-IκBα, IκBα, phospho-IKKα/β, β-actin, and the horseradish peroxidase-conjugated goat anti-rabbit secondary antibody were purchased from Cell Signaling Technology, Inc. (Danvers, MA, United States). The antibodies specific for phospho-cPLA_2_, NF-κB p65, lamin B, LAT, Lyn, Fyn, and Syk, as well as Bay 61-3606 reagent were obtained from Santa Cruz Biotechnology, Inc. (Dallas, TX, United States). The LTC_4_ enzyme linked immunoassay (EIA) kit, and the antibody for COX-2 were from Cayman Chemical (Ann Arbor, MI, United States). Histamine ELISA kit was purchased from Demeditec Diagnostics GmbH (Kiel, Germany).

### Plant Material

Spinacetin was isolated from the ethanol extract of the *I. japonica* (Supplementary Material). The plants of *I. japonica* were collected from Henan Province, China, and identified by Professor Y. Zhou (Department of Pharmacognosy, School of Pharmacy, Tianjin Medical University). A voucher specimen (IJ201105) was deposited at School of Pharmacy, Tianjin Medical University, China. The flower part of *I. japonica* was used to isolate spinacetin. Prior to use, spinacetin was dissolved in dimethyl sulfoxide (DMSO).

### Cell Culture

Bone marrow-derived mast cells were isolated from bone marrow of Balb/c mice and differentiated as described by us previously (Jin et al., 2017). Briefly, bone marrow cells from Balb/c mice were cultured in RPMI1640 containing 0.1 mM non-essential amino acid solution, 100 U/ml of penicillin, 100 μg/ml of streptomycin, 10% FBS and 20% pokeweed mitogen-stimulated spleen conditioned medium as a source of IL-3. The differentiated cells were available for use after 3 weeks, when more than 99% were found to become BMMCs as checked with the method previously described (Murakami et al., 1994). HMC-1 was kindly provided by Dr. Joseph Butterfield (Mayo Clinic, Rochester, MN, United States) and RBL-2H3 cells was purchased from the Cell Bank of Chinese Academy of Sciences (Shanghai, China). HMC-1 and RBL-2H3 cells were cultured in IMDM (Iscove’s modified Dulbecco’s medium) and DMEM (Dulbecco’s Modified Eagle’s Medium) supplemented with 10% FBS without IL-3, respectively.

### Activation of BMMCs, RBL-2H3, and HMC-1

Bone marrow-derived mast cells and RBL-2H3 were sensitized with 500 ng/ml of anti-DNP IgE overnight, and then treated with spinacetin or Bay 61-3606 for 1 h. After stimulated with 100 ng/ml of DNP-HSA (Ag) for 15 min or 6 h, the supernatants were collected for further analysis, respectively. The levels of LTC_4_, histamine and IL-6 were determined using an immunoassay kit according to the manufacturer’s protocol. The HMC-1 cells were incubated with spinacetin in the presence or absence of phorbol 12-myristate 13-acetate (PMA) and calcium ionophore (A23187).

### Measurement of Intracellular Ca^2+^ Level

Intracellular Ca^2+^ level was determined with the FluoForte Calcium Assay Kit (Enzo Life Sciences, Ann Arbor, MI, United States) as described previously (Jin et al., 2017). Briefly, IgE-sensitized BMMCs were pre-incubated with FluoForte^TM^ dye-loading solution for 1 h, and then treated with spinacetin or Bay 61-3606 for 1 h. After addition of DNP-HSA and incubation for 5 min, the fluorescence was monitored with a multilabel plate reader at Ex = 485 nm/Em 535 nm (Perkin Elmer’s VICTOR^TM^*X*5 Multilabel Plate Reader, Waltham, MA, United States).

### Extraction of Nuclear Proteins

Nuclear extracts were prepared as described before. Briefly, IgE-sensitized BMMCs and RBL-2H3 cells were pretreated with spinacetin for 1 h, followed by incubation with DNP-HSA for 30 min. Then the nuclear proteins were prepared by using Nuclear Extraction Kit according to the manufacturer’s protocol (Panomics, Fremont, CA, United States).

### Western Blot Analysis

IgE-sensitized BMMCs or RBL-2H3 were pretreated with spinacetin or Bay 61-3606 for 1 h and stimulated with DNP-HSA. The preparation of cell lysates and Western blot were performed as described previously (Wang et al., 2015). To assess COX-2 expression, BMMCs were pre-incubated with 1 μg/ml aspirin for 2 h to irreversibly inactivate preexisting COX-1. In the case of HMC-1, cells were pretreated with spinacetin for 1 h, stimulated with PMA and A23187, then collected for preparation of the cell lysates.

### Immunoprecipitation (IP)

Total cell lysates were incubated with anti-Syk, anti-LAT, anti-Fyn or anti-Lyn antibody overnight, and immune-complexes were precipitated with protein A/G plus agarose. Precipitates were subjected to SDS-PAGE and immunoblotted with the respective antibodies.

### PCA

ICR mice (7 weeks old male) were intradermally injected with 80 ng of mouse anti-dinitrophenyl (DNP) IgE (Sigma) into one ear. After 24 h, spinacetin or dexamethasone (Dexa) was orally administered. One hour later, the mice were intravenously challenged with 60 μg of DNP-HSA in 200 μl of PBS containing 1% (w/v) Evans blue. The mice were euthanized 1 h after treatment with DNP-HSA. The ears were removed, and then dissolved with 400 μl of formamide at 63°C overnight to measure the amount of dye extravasated by Ag. The absorbance of dye was measured at 630 nm using a microplate reader (BIO-RAD iMark, Hercules, CA, United States). The ears were fixed with 4% formaldehyde and embedded in paraffin. Four micrometer sections of the tissues were prepared and stained with toluidine blue to count the number of mast cells. All experiments using animals were approved on Oct 27, 2017, by the Institutional Animal Care and Use Committee of Tianjin Medical University (No. of allowance: TMUaMEC2017022).

### Statistical Analysis

All values are expressed as means ± SEM of triplicate values. One-way ANOVA was utilized to determine the statistical significance with GraphPad Prism 7 (GraphPad Software, La Jolla, CA, United States). Differences were considered statistically significant when *P* < 0.05.

## Results

### Spinacetin Suppresses Histamine Release and Ca^2+^ Mobilization in IgE/Ag-Stimulated BMMCs

The MTT assay was employed to determine the cell viability of spinacetin against BMMCs. As shown in **Figure 1B**, spinacetin was not found to affect cell viability at concentration of less than 5 μM. Thus, concentrations of 1, 2, and 5 μM were chosen for further study to assess the anti-inflammatory mechanism.

Fc𝜀RI aggregation is known to stimulate mast cell release of preformed mediators, including histamine, protease, and proteoglycans (Kopec et al., 2006). And histamine is a crucial mediator in the inflammation and allergic response (Gibbs and Levi-Schaffer, 2012; Cataldi et al., 2014). Therefore, we investigated the inhibitory effect of spinacetin on histamine release in IgE/Ag-stimulated BMMCs. As shown in **Figure 1C**, spinacetin strongly inhibited release of histamine. Increased cytosolic Ca^2+^ mobilization leads to mast cell degranulation and production of various pro-inflammatory mediators (Kopec et al., 2006). Thus, we examined the effect of spinacetin on intracellular Ca^2+^ mobilization. As shown in **Figure 1D**, intracellular Ca^2+^ level was potently increased in activated BMMCs, and this increase was attenuated by spinacetin. The Syk inhibitor Bay 61-3606 was used as positive control. These data indicate that spinacetin inhibits mast cell degranulation through suppression of Ca^2+^ mobilization.

### Spinacetin Inhibits LTC_4_ Generation via Blocking MAPKs Phosphorylation and cPLA_2_ Translocation Into the Nucleus

**FIGURE 2:**
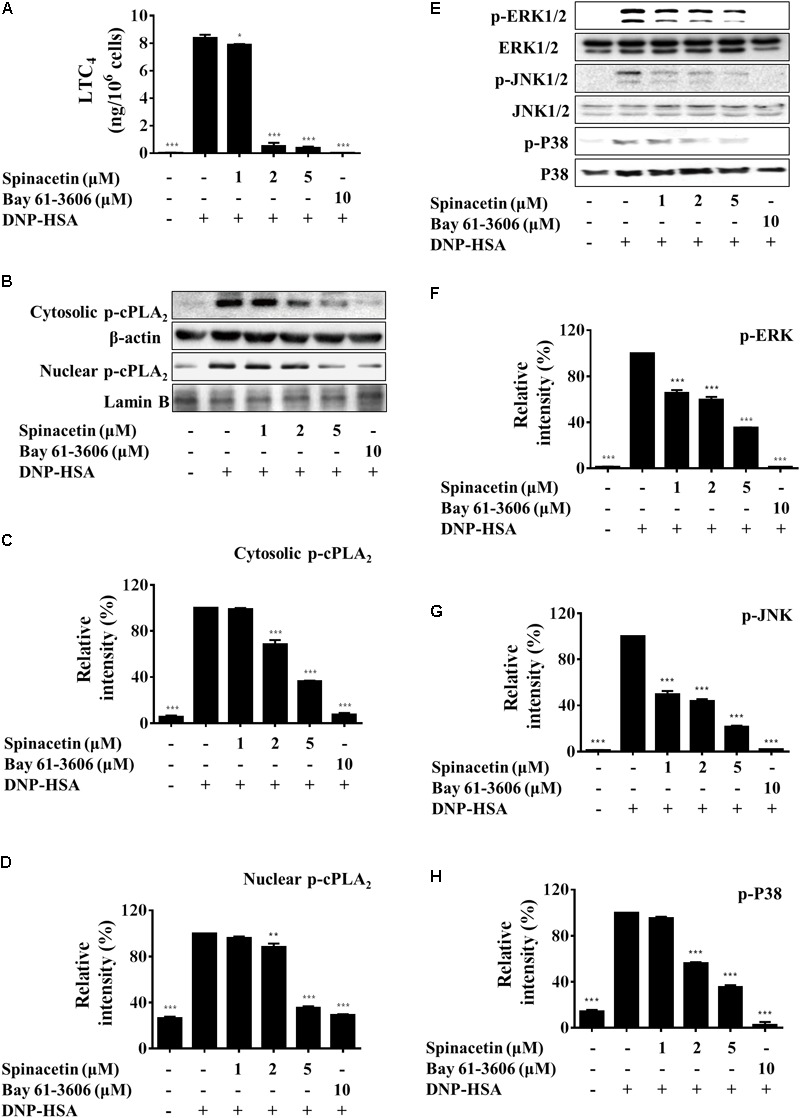
Spinacetin suppresses LTC_4_ generation, cPLA_2_ translocation, and MAPKs phosphorylation. IgE-sensitized BMMCs were pre-incubated with indicated concentrations of spinacetin or Bay 61-3606 for 1 h, then stimulated with DNP-HSA for 15 min. **(A)** The culture medium was collected and measured for generation of LTC_4_. **(B)** Cytosolic and nuclear fractions were immunoblotted with antibody for phospho-cPLA_2_, and relative ratios of cytosolic p-cPLA_2_ **(C)**, nuclear p-cPLA_2_ **(D)** proteins were determined by analyzing immunoblot band intensities. **(E)** Cell lysates were immunoblotted for the phosphorylated and total forms of ERK1/2, JNK1/2, and p38. Relative ratios of cytosolic p-ERK1/2/ERK1/2 **(F)**, p-JNK1/2/JNK1/2 **(G)**, and p-p38/p38 **(H)** proteins were determined by analyzing immunoblot band intensities. The results show the mean ± SEM of three independent experiments. ^∗^*P* < 0.05, ^∗∗^*P* < 0.01, and ^∗∗∗^*P* < 0.001, compared with the cells with IgE/Ag stimulation but without spinacetin treatment.

Leukotrienes are a family of potent eicosanoid lipid mediators with key role in the pathogenesis of several inflammatory diseases like asthma (Duroudier et al., 2009). When activated by IgE/Ag, mast cells mobilize arachidonic acid through cPLA_2_, and rapidly synthesize LTC_4_ (Jin et al., 2017). As shown in **Figure 2A**, after spinacetin treatment, the generation of LTC_4_ was significantly decreased in a dose-dependent manner in IgE/Ag-stimulated BMMCs. IgE/Ag stimulation potently increased cPLA_2_ phosphorylation and promoted its nuclear translocation, while this increased activation was down-regulated by spinacetin (**Figures 2B–D**). The enzymatic activity of cPLA_2_ is known to be increased by phosphorylation of MAPKs (Lin et al., 1993). Therefore, next we assessed the effect of spinacetin on phosphorylation of MAPKs. As shown in **Figures 2E–H**, the IgE/Ag-induced phosphorylation of ERK1/2, JNK1/2 and p38 was significantly decreased by spinacetin. The above results indicated that inhibition of ERK1/2, JNK1/2, and p38 by spinacetin was involved in blockade of cPLA_2_ activation and the subsequent LTC_4_ synthesis.

### Spinacetin Decreases IL-6 and COX-2 Expression by Suppressing Akt/IκBα/NFκB Pathway

**FIGURE 3:**
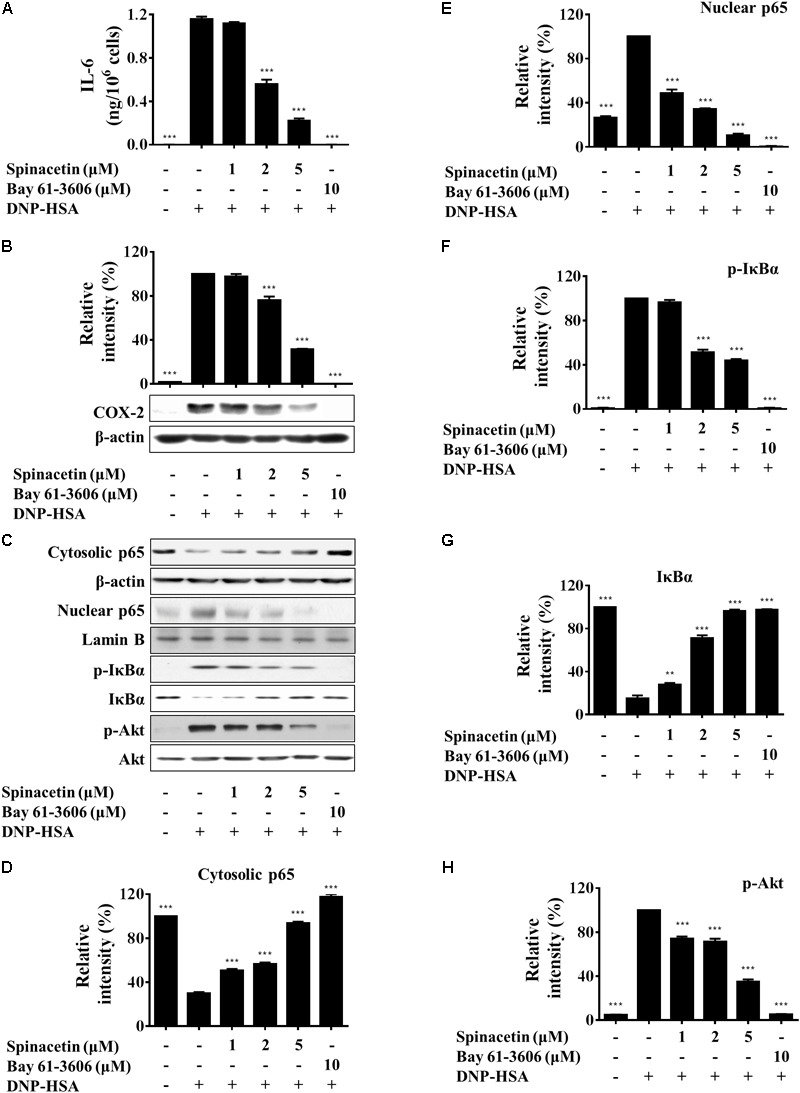
Spinacetin inhibits production of IL-6 and expression of COX-2 through down-regulation of Akt/IκB/NFκB pathway. **(A)** IgE-sensitized BMMCs were pre-treated with different concentrations of spinacetin for 1 h and stimulated with DNP-HSA for 6 h. The supernatants were collected to determine the levels of IL-6 by ELISA. **(B)** IgE-sensitized BMMCs were pretreated with aspirin for 2 h, treated with spinacetin for 1 h, and then stimulated with DNP-HSA for 7 h. The immunoblot was then probed with anti-COX-2 antibody. **(C)** IgE-sensitized BMMCs were pre-incubated with spinacetin for 1 h and then stimulated with DNP-HSA for an additional 15 min. Cell lysates of cytosolic and nuclear proteins, and total cell lysates were examined with western blot analysis for p65, p-IκBα, IκBα, and p-Akt. **(D–H)** Relative ratios of the respective proteins were determined by analyzing immunoblot band intensities. The results show the mean ± SEM of three independent experiments. ^∗∗^*P* < 0.01 and ^∗∗∗^*P* < 0.001, compared with the cells with IgE/Ag stimulation but without spinacetin treatment.

Interlukin-6 is a pleiotropic cytokine and plays an important role in inflammatory diseases (Yao et al., 2014). Therefore, we examined the effect of spinacetin on IL-6 production in IgE/Ag-stimulated BMMCs. **Figure 3A** shows that spinacetin treatment inhibited level of IL-6 dose dependently. COX-2 is an important enzyme regulating production of PGs, which are important mediators in inflammation (Tsatsanis et al., 2006). In order to abolish preexisting COX-1, BMMCs were pre-treated with aspirin for 2 h. Then the cells were stimulated with DNP-HSA for 7 h in the absence or presence of spinacetin. As shown in **Figure 3B**, the COX-2 protein was not detected in unstimulated BMMCs. Stimulation with IgE/Ag potently induced expression of COX-2, which was reduced by spinacetin in a dose-dependent manner.

Nuclear factor-κB positively regulates the production of IL-6 and COX-2 expression (Tsatsanis et al., 2006; Ponce et al., 2009). Activation of NF-κB, an essential transcription factor involved in the inflammatory responses, occurs following the phosphorylation, ubiquitination and degradation of IκBα. Then activated NF-κB translocates from cytosol into the nucleus (Chen et al., 1999). RelA (p65) is a subunit of NF-κB (Ghosh and Karin, 2002). In order to study whether spinacetin decreases the generation of IL-6 through inactivation of NF-κB or not, we investigated the effect of spinacetin on NF-κB. As shown in **Figures 3C–E**, p65 translocated from cytosol to the nucleus after stimulation with IgE/Ag. However, spinacetin significantly inhibited p65 translocation. Furthermore, spinacetin treatment suppressed phosphorylation and degradation of IκBα (**Figures 3C,F,G**). NF-κB is known to be activated by PI3K/Akt pathway (Manukyan et al., 2010; Shi et al., 2016). IgE/Ag stimulation resulted in increase of the phosphorylated Akt, and spinacetin inhibited this activity (**Figures 3C,H**). Our results suggested that inhibition of Akt by spinacetin might be involved in suppression of IL-6 and COX-2 expression.

### Spinacetin Inhibits RBL-2H3 Activation but Does Not Affect HMC-1

**FIGURE 4:**
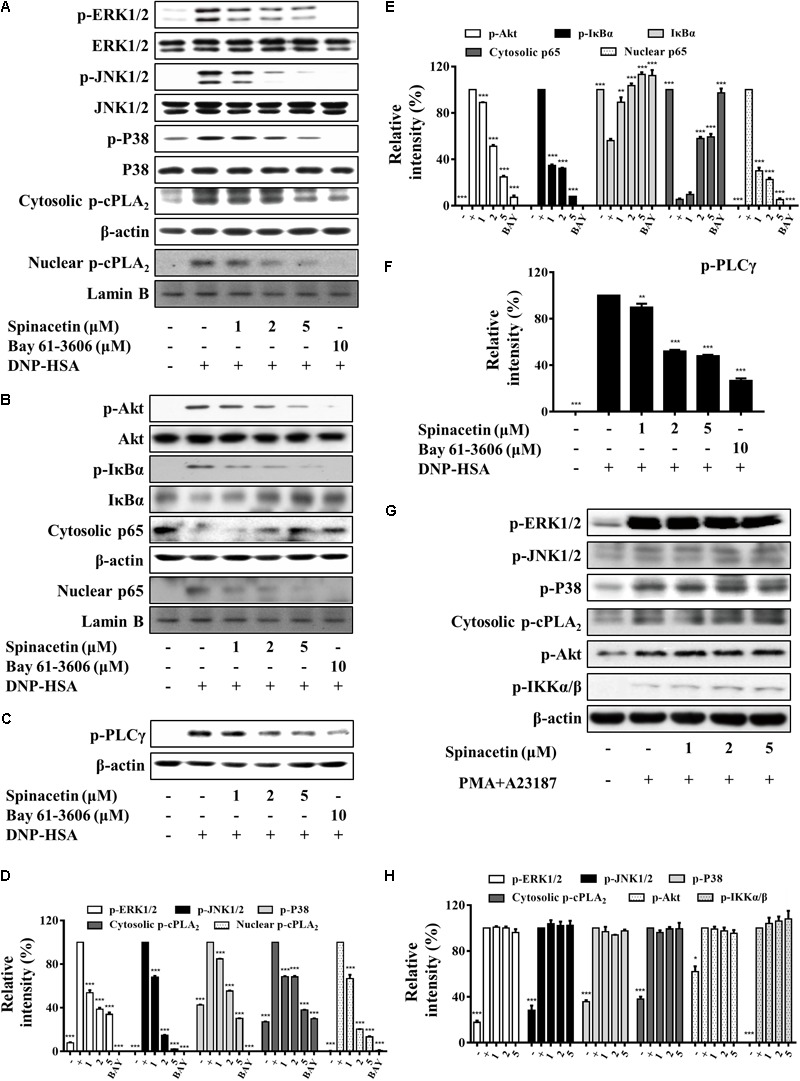
Spinacetin attenuates activation of RBL-2H3 without effect on HMC-1 cells. IgE-sensitized RBL-2H3 cells were pre-incubated with spinacetin for 1 h and then stimulated with DNP-HSA for 15 min. Cell lysates were immunoblotted for the phosphorylated and total forms of ERK1/2, JNK1/2, p38, and cytosolic and nuclear p-cPLA_2_ **(A,D)**, p-Akt, p-IκBα, IκBα, cytosolic and nuclear NF-κB p65 **(B,E)**, and p-PLCγ **(C,F)**. Relative intensities of the respective signal proteins were determined by using ImageJ software. The results show the mean ± SEM of three independent experiments. “–”: RBL-2H3 cells sensitized with IgE; “+”: RBL-2H3 cells sensitized with IgE/DNP-HSA. Bay: Bay 61-3606. **(G)** HMC-1 cells were pre-treated with spinacetin for 1 h, then stimulated with PMA plus A23187 for 15 min. HMC-1 cells lysates were prepared for Western blot of p-ERK1/2, p-JNK1/2, p-p38, p-cPLA_2_, p-Akt, and IKKα/β proteins. **(H)** Relative intensities of the respective signal proteins in **(G)** were determined by using ImageJ software. The results show the mean ± SEM of three independent experiments. “–”: non-treated HMC-1 cells; “+”: HMC-1 cells sensitized with PMA/A23187. ^∗^*P* < 0.05, ^∗∗^*P* < 0.01, and ^∗∗∗^*P* < 0.001, compared with the cells with IgE/Ag or PMA/A23187 stimulation but without spinacetin treatment.

RBL-2H3 cells are widely used for *in vitro* studies of mast cell-mediated allergic inflammation. Stimulation of RBL-2H3 with IgE/Ag increased ERK1/2, JNK1/2, and p38 phosphorylation as compared to non-treated cells (**Figures 4A,D**). Spinacetin reduced the activation of the MAPKs. Meanwhile, spinacetin markedly reduced translocation of the phosphorylated cPLA_2_ from cytosol into the nucleus (**Figures 4A,D**). Moreover, spinacetin suppressed IκBα phosphorylation and degradation, and NF-κB activation, and also inhibited Akt which is known to activate IκBα and NF-κB (**Figures 4B,E**). These results are consistant with that obtained in IgE/Ag-stimulated BMMCs. Furthermore, the phosphorylation of PLCγ was inhibited by spinacetin (**Figures 4C,F**). These results demonstrate that spinacetin ameliorated the allergic reaction in IgE/Ag stimulated mast cells.

HMC-1 cells are characterized to lack expression of Fc𝜀RI. Therefore, many researchers use calcium ionophores and phorblo esters to measure mast cell activity by using HMC-1 cells. HMC-1 can be activated by PMA and A23187, and the activated HMC-1 leads to the phosphorylation of tyrosine kinase followed by the activation of MAPKs, NF-κB, and release of pro-inflammatory cytokines (Park et al., 2014). In our study, HMC-1 cells were pre-treated with spinacetin for 1 h, and then stimulated with PMA and A23187. However, spinacetin did not suppress phosphorylation of MAPKs, cPLA_2_, Akt and IKKα/β in HMC-1 cells (**Figures 4G,H**).

These results suggest that spinacetin only inhibits IgE/Ag-induced mast cells activation, and that target protein of spinacetin might be upstream of Akt.

### Spinacetin Inhibits PLCγ, LAT, and Syk Phosphorylation Independent of Lyn and Fyn

**FIGURE 5:**
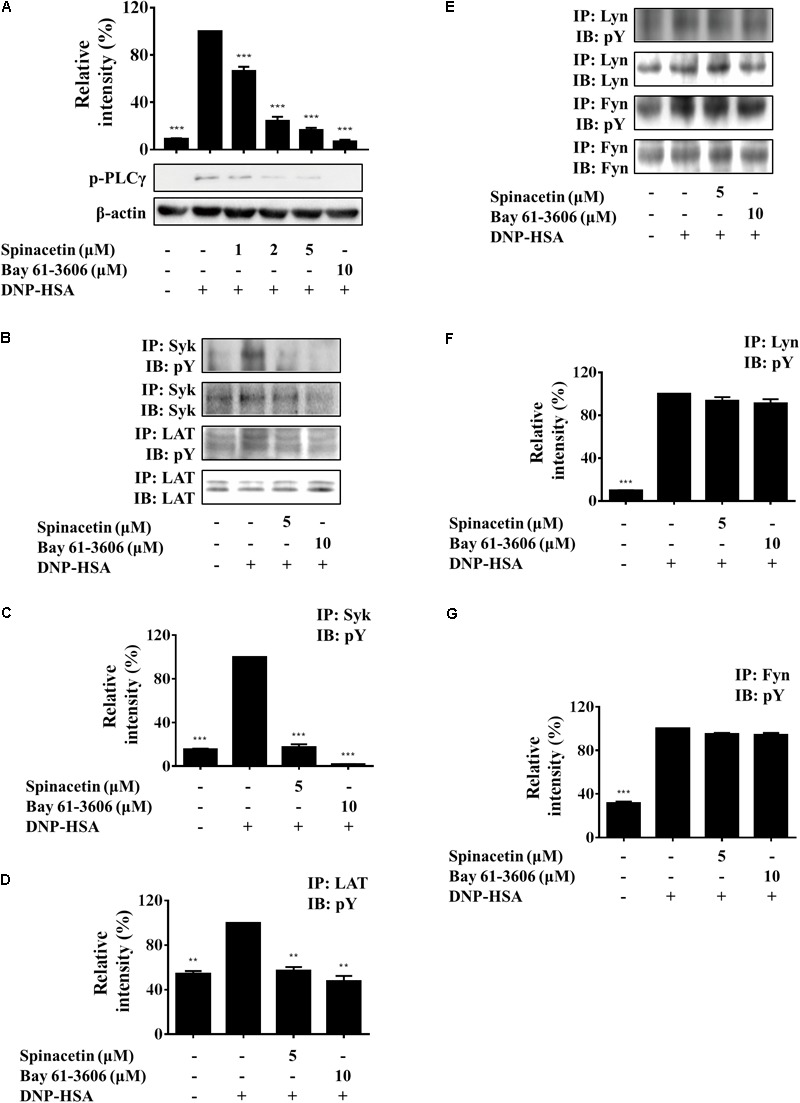
Spinacetin inhibits the Syk-associated pathway. IgE-sensitized BMMCs were pre-incubated with spinacetin for 1 h and then stimulated with DNP-HSA for 5 min. Cell lysates were subjected to immunoblot and immunoprecipitation analysis for the phosphorylated forms of PLCγ **(A)**, Syk and LAT **(B)**, Lyn and Fyn **(E)**. **(C,D,F,G)** Relative ratios of several proteins were determined by analyzing immunoblot band intensities. The data show the mean ± SEM of three independent experiments. ^∗∗^*P* < 0.01 and ^∗∗∗^*P* < 0.001, compared with the cells with IgE/Ag stimulation but without spinacetin treatment.

Lyn phosphorylates and activates Syk and LAT. Phosphorylation of LAT results in the recruitment of molecules such as Grb2, Gads, SOS, and PLCγ. These interactions with LAT lead to the formation of molecular signaling complex that is required for the release and generation of pro-inflammatory mediators in mast cells (Gilfillan and Tkaczyk, 2006). Fyn is also required for mast cell degranulation, cytokine, and chemokine production (Kopec et al., 2006). Fyn phosphorylates Gab2 to activate PI3K upon Fc𝜀RI aggregation (Rivera and Gilfillan, 2006). Activated PLCγ induces mobilization of cytosolic Ca^2+^ and activates PKC, respectively (Kopec et al., 2006). As shown in **Figure 5A**, Ag stimulation increased phosphorylation of PLCγ, and spinacetin remarkably reduced phosphorylated PLCγ. Treatment with spinacetin inhibited the histamine release, LTC_4_ synthesis and IL-6 production, which are critical for allergic inflammation. And generation of the pro-inflammatory mediators was controlled by Ca^2+^ mobilization, cPLA_2_, MAPK, NF-κB, and PLCγ, respectively. Therefore, next we investigated whether spinacetin affects the upstream molecules such as LAT, Syk, Lyn, and Fyn. As shown in **Figures 5B–D**, spinacetin inhibited phosphorylation of LAT and Syk. However, spinacetin did not affect Lyn and Fyn (**Figures 5E–G**). These observations indicated that spinacetin inhibited the IgE-mediated mast cell activation through regulating Syk dependent pathways.

### Spinacetin Attenuates IgE-Mediated PCA

**FIGURE 6:**
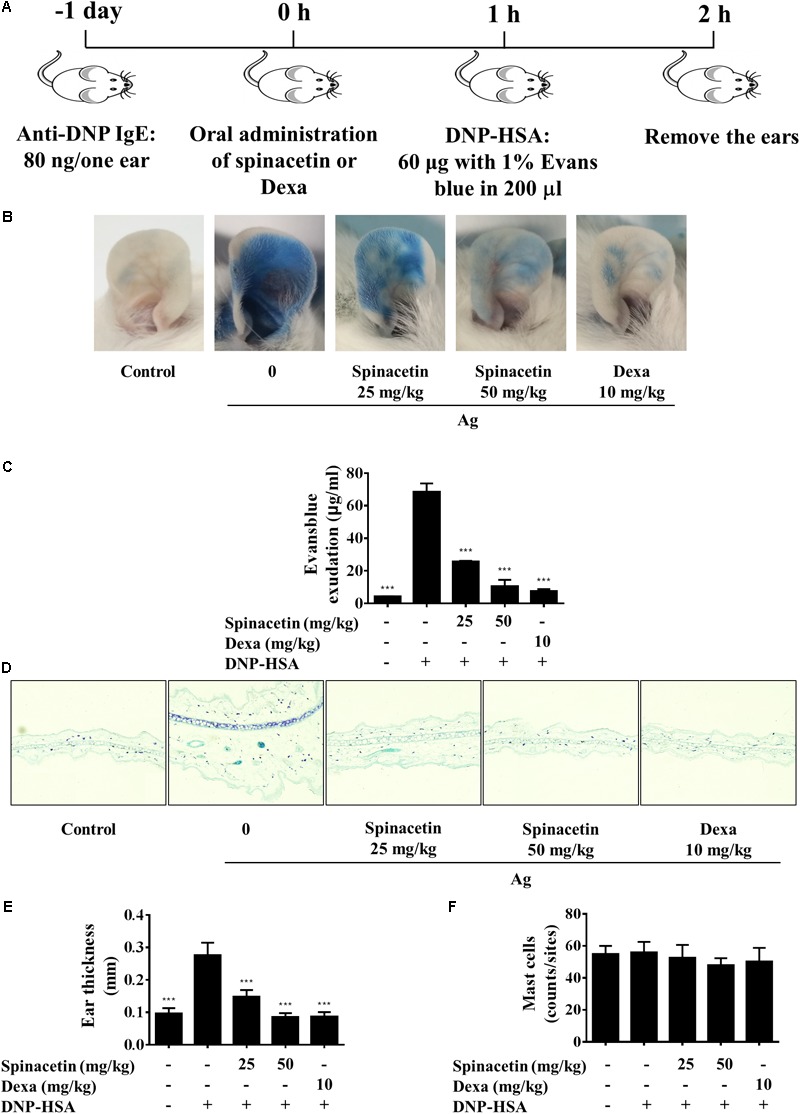
Spinacetin inhibits IgE-mediated PCA reaction. **(A)** ICR mice were injected intradermally with 80 ng of anti-DNP IgE into one ear. After 24 h, the mice were intravenously injected with 60 μg of DNP-HSA containing 1% Evans blue. Spinacetin was orally administered 1 h before Ag administration. **(B,C)** The dye extracted from per ear was detected using spectrophotometer. **(D,E)** Representative photomicrographs of ear sections were stained with toluidine blue, and ear thickness was measured. **(F)** The number of mast cells was counted at the dermis. The data show the mean ± SEM (*n* = 5 per group). ^∗∗∗^*P* < 0.001, compared with the mice with IgE/Ag sensitization but without drug treatment.

The Fc𝜀RI/mast cell axis is involved in triggering several intracellular signaling molecules to release preformed granules, and to generate pro-inflammatory mediators, resulting in the induction of allergy and anaphylaxis (Manikandan et al., 2012). As we already found that spinacetin had shown potent anti-inflammatory activity in IgE/Ag-stimulated mast cells, therefore we assessed the anti-allergic effects of spinacetin by using a PCA model in mice. PCA was measured by intravenously injection with Ag (DNP-HSA in 1% Evans blue dye) to sensitized mice after oral administration of 25 and 50 mg/kg spinacetin or Dexa (**Figure 6A**). Dexa was used as positive drug. As shown in **Figure 6**, IgE/Ag significantly induced PCA reaction, and treatment with spinacetin markedly decreased PCA reaction which is reflected by amount of diffused dye (**Figures 6B,C**) and ear thickness (**Figures 6D,E**), compared to the Ag challenged group. These attenuated allergic reactions might be due to the inhibitory effect of spinacetin on activation of mast cells. To confirm whether the decreased vascular permeability was related to amount of mast cells, the number of mast cells was counted at the ear reaction site. There was no variation between Ag treated group and spinacetin treated group (**Figure 6F**).

## Discussion

Flavonoids are abundant in plants, with a variety of biological functions such as anticancer, antiviral, antibacterial, anti-oxidative, and anti-inflammatory activities (Panche et al., 2016; Zakaryan et al., 2017). Particularly, anti-inflammatory activities of flavonoids were frequently reported. Luteolin suppresses inflammation-associated gene expression by blocking NF-κB and activating protein-1 (AP-1) activation (Chen et al., 2007). Quercetin reduces lipopolysaccharide (LPS)-induced inflammation via inhibition of PI3K (Endale et al., 2013). Wogonin attenuates inflammation by activating PPAR-γ (Li et al., 2017). However, it has been rarely reported about the bioactivities of spinacetin. Recently, we isolated spinacetin from *Inula japonica* Thunb. In the present study, we report its anti-inflammatory effect on IgE/Ag-induced mast cells and PCA mouse model as well as the related molecular mechanism.

Mast cells can function as effector cells in certain innate and adaptive immune responses (Galli et al., 2008). These cells express high affinity Fc𝜀RI on the cell surface which strongly binds IgE antibodies (Kinet, 1999). Fc𝜀RI crosslinking induces mast cell degranulation (granules include histamine, proteoglycans, and proteases) and production of several pro-inflammatory mediators including LTC_4_, IL-6, TNFα, and enzyme COX-2 (Lu et al., 2011; Moon et al., 2014; Jin et al., 2017). Release of LTs has been implicated in inflammation and allergy (Henderson, 1994). Histamine also plays an important role in allergic disease such as asthma, rhinitis, urticaria, and anaphylaxis (White, 1990). IL-6 level was elevated in numerous inflammatory diseases such as rheumatoid arthritis, systemic lupus erythematosus and psoriasis, suggesting its close involvement in inflammation (Gabay, 2006). In the present study, we found that the mediators that BMMCs generated, including histamine, LTC_4_, IL-6, and COX-2, were inhibited by spinacetin (**Figures 1C, 2A, 3A,B**).

Aggregation of the high-affinity IgE receptor Fc𝜀RI results in exocytosis of granules and production of LTs and cytokines. After binding of antigen to IgE/Fc𝜀RI complexes, Lyn transphosphorylates the ITAMs of β- and γ- chains of Fc𝜀RI, which are associated with the Src family tyrosine kinases Lyn and Fyn and with the non-Src tyrosine kinase Syk (Geldman and Pallen, 2015). Fyn, Lyn and Syk contribute to the formation of multimolecular signaling complexes that are coordinated by several adaptor molecules like LAT, Gads, Grb2, Gab2, and signaling proteins like PLCγ (Rivera et al., 2008). Fc𝜀RI -mediated PLCγ activation results in generation of IP_3_, which binds to its receptor on the endoplasmic reticulum leading to liberation of Ca^2+^ from intracellular store. We found that spinacetin suppressed mobilization of Ca^2+^ and phosphorylation of PLCγ (**Figures 1D, 5A**). The activated Vav, Grb2, and SOS lead to phosphorylation of ERK, JNK, and p38, and activation of cPLA_2_ to release arachidonic acid (Siraganian, 2003). In the present study, we have clearly shown that spinacetin suppressed LTC_4_ generation through cPLA_2_ phosphorylation and translocation, associated with MAPKs activation (**Figures 2B–D**).

Crosslinking of Fc𝜀RI-bound IgE-molecules with multivalent antigen or allergen induces NF-κB activation (Klemm and Ruland, 2006). NF-κB is a nuclear transcription factor involved in various biological processes such as inflammation and tumorigenesis (Ghosh and Dass, 2016). Therefore, the involvement of spinacetin in IgE/Ag-induced activation of NF-κB was investigated. Spinacetin potently inhibited phosphorylation of Akt, phosphorylation and degradation of IκBα, and finally decreased p65 translocation (**Figures 3C–H**). These findings suggest that spinacetin effectively inhibits IgE/Ag-induced NF-κB activation, in which suppression of Akt phosphorylation might be involved.

We also observed spinacetin potently inhibits IgE/Ag-induced activation of RBL-2H3 cells (**Figures 4A–F**). These findings are consistent with down-regulation of ERK1/2, JNK1/2, p38 MAPKs, and Akt/IκB/NF-κB signal by spinacetin in IgE/Ag-stimulated BMMCs. However, spinacetin did not affect the corresponding molecules in PMA and A23187-induced HMC-1 cells, including ERK1/2, JNK1/2, p38 MAPKs, and Akt/IKKα/β (**Figures 4G,H**). It is known that there is no expression of Fc𝜀RI on HMC-1 surface. These results suggest that spinacetin does not affect Akt, MAPKs, and their downstream proteins, and target protein of spinacetin may be in the upstream of Akt.

In order to know whether spinacetin targeted any protein upstream of Akt, we investigated the effect of spinacetin on phosphorylation of PLCγ, LAT, Syk, Lyn, and Fyn. As a result, spinacetin inhibited phosphorylation of PLCγ, LAT, and Syk (**Figures 5A–D**). However, it did not affect Lyn and Fyn (**Figures 5E–G**). These results indicate that spinacetin inhibits Syk-dependent pathway in IgE/Ag-induced mast cells.

Anaphylaxis is the most serious allergic reaction, and typically occurs from the activation of mast cells and basophils through crosslinking of IgE and aggregation of Fc𝜀RI (Peavy and Metcalfe, 2008; Guilarte et al., 2017). In order to investigate whether spinacetin attenuates allergic response *in vivo*, we used a mast cell-dependent PCA model. The oral administration of spinacetin 1 h prior to challenge with Ag effectively impaired extravasation of Evans blue dye in the ear, and ear thickness (**Figure 6**), suggesting the *in vivo* anti-anaphylaxis efficacy of spinacetin.

**FIGURE 7:**
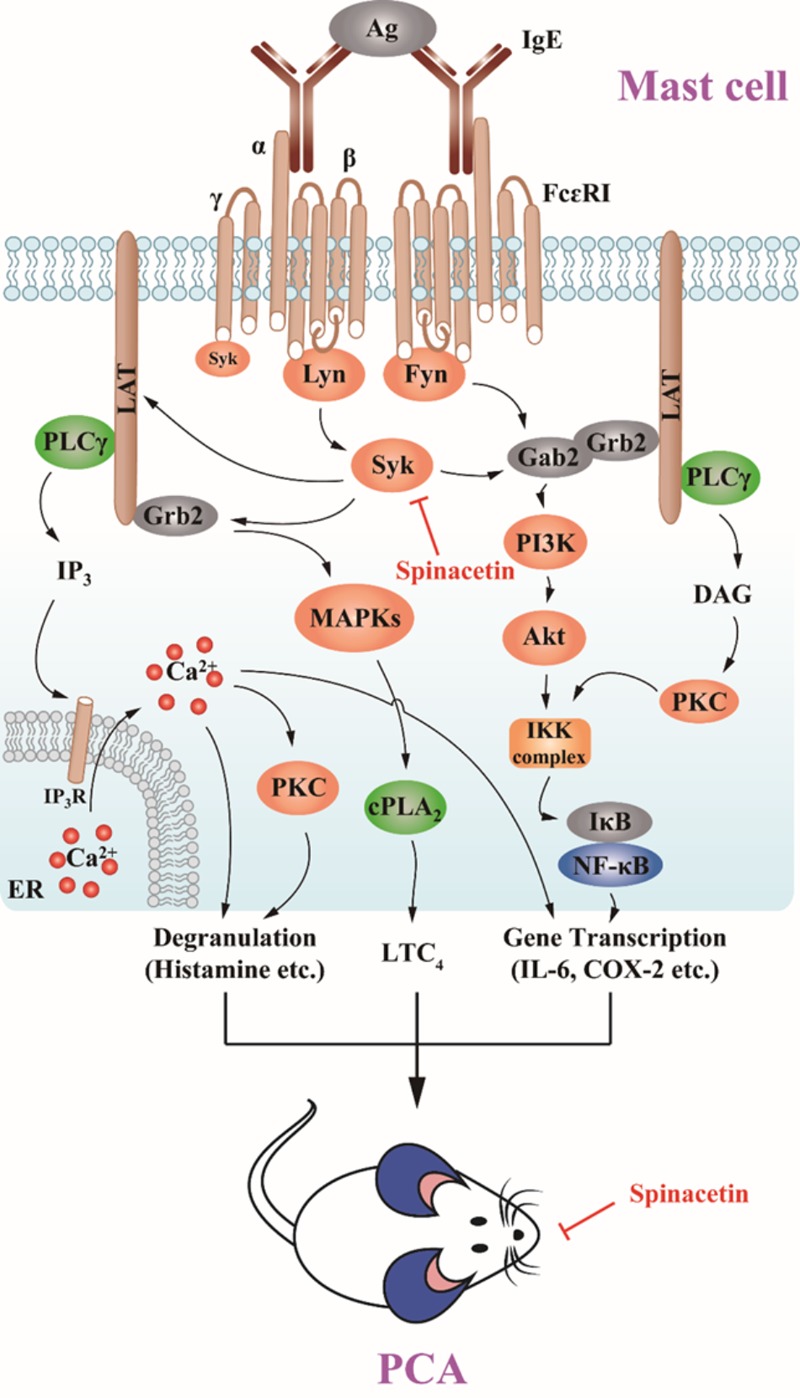
Spinacetin suppresses the IgE-mediated activation of mast cell and PCA reaction.

In the present study, we demonstrated that spinacetin inhibited Syk, LAT, PLCγ activation, subsequently down-regulated Akt, IκBα, NF-κB, MAPKs, cPLA_2_, and Ca^2+^ mobilization, finally suppressed histamine release, LTC_4_ synthesis, and IL-6 production in IgE/Ag-stimulated BMMCs (**Figure 7**). Spinacetin also inhibited the corresponding signals in IgE/Ag-induced RBL-2H3 cells, but did not affect those in PMA and A23187-induced HMC-1 cells. Furthermore, spinacetin attenuated IgE/Ag-mediated PCA reaction in mouse model. This study suggests that spinacetin could be used as a lead compound for development of novel anti-inflammatory drugs.

## Author Contributions

NJ, SP, CS, and YC performed the experiments. ZZ, RW, and YQ provided technical assistances. NJ and MJ wrote the manuscript. MJ and DK designed the experiments and acquired funding for the study. DK edited the manuscript.

## Conflict of Interest Statement

The authors declare that the research was conducted in the absence of any commercial or financial relationships that could be construed as a potential conflict of interest.

## Abbreviations

BMMCs: bone marrow-derived mast cells
cPLA_2_: cytosolic phospholipase A_2_
ERK1/2: extracellular signal regulated-protein kinase 1/2
IL-6: interleukin-6
JNK1/2: c-Jun-NH_2_-terminal kinase 1/2
LAT: linker of activated T cells
LTC_4_: leukotriene C_4_
MAPKs: mitogen-activated protein kinases
NF-κB: nuclear factor-κB
PCA: passive cutaneous anaphylaxis
PLCγ: phospholipase Cγ
