# Supplementary material for: Spinacetin Suppresses the Mast Cell Activation and Passive Cutaneous Anaphylaxis in Mouse Model
Source: Front Pharmacol. 2018 Jul 30;9:824. doi: 10.3389/fphar.2018.00824 (PMC6077219; doi:10.3389/fphar.2018.00824)
Supplement: Supplementary file 1 [file Presentation_1.PDF]

Supplementary Material

Spinacetin suppresses the mast cell activation and passive cutaneous anaphylaxis in mouse model

Ning Ji [1], Shunli Pan [1], Chen Shao [1], Yufen Chen [1, 2], Zhe Zhang [1], Ran Wang [1], Yuling Qiu [1], Meihua Jin [1*] and Dexin Kong[1*]

[1]*Tianjin Key Laboratory on Technologies Enabling Development of Clinical Therapeutics and Diagnostics, School of Pharmacy, Tianjin Medical University, Tianjin 300070,China;* [2]*Pharmacy Department, Tanggu Hospital of Infectious Diseases of Tianjin Binhai New Area, Tianjin 300454, China*

[*]**Correspondence** Meihua Jin, jinmeihua@tmu.edu.cn; Dexin Kong kongdexin@tmu.edu.cn

MATERIALS AND METHODS

Extraction and isolation of spinacetin

The powdered and dried flowers of *I. japonica* (8.0 kg) were extracted with 75% ethanol under reflux. The extract was concentrated to give a residue (600 g), which was suspended in water and partitioned with petroleum ether (PE), ethyl acetate (EtOAc) and *n*-butyl alcohol (*n*-BuOH), successively.

The EtOAc-partitioned extract (117 g) was subjected to column chromatography and semi-preparative HPLC (high performance liquid chromatography) successively to afford compound spinacetin (55 mg).

The isolated compound was identified as spinacetin by comparing the MS and NMR data with those reported (Bai et al., 2005).

REFERENCES

Bai, N., Zhou, Z., Zhu, N., Zhang, L., Quan, Z., He, K., et al. (2005). Antioxidative flavonoids from the flower of *Inula britannica*. *J Food Lipids* 12(2)**,** 141-149. doi: doi:10.1111/j.1745-4522.2005.00012.x.
